# Ectopic Intramural Isthmic Pregnancy: Case Report

**DOI:** 10.3390/jcm14145146

**Published:** 2025-07-20

**Authors:** Eloisa Maria Mariani, Diletta Guglielmi, Paola Camponovo, Erika Gambino, Alessandra Inzoli, Davide Leni, Paolo Passoni, Anna Locatelli

**Affiliations:** 1 Obstetrics and Gynecology, IRCCS San Gerardo dei Tintori Foundation, 20900 Monza, Italy; eloisamaria.mariani@irccs-sangerardo.it (E.M.M.); a.inzoli2@campus.unimib.it (A.I.);; 2 Specialization School in Obstetrics and Gynecology, University of Milano-Bicocca, 20100 Monza, Italy; 3 Department of Diagnostic Radiology, Fondazione IRCCS San Gerardo dei Tintori, 20900 Monza, Italy; 4 School of Medicine and Surgery, University of Milano-Bicocca, 20100 Monza, Italy

**Keywords:** Methotrexate (MTX), fertility preservation, intramural pregnancy, ectopic pregnancy (EP), uterine artery embolization (UAE)

## Abstract

**Background/Objectives:** Intramural pregnancy (IMP) is a rare type of ectopic pregnancy where the embryo implants within the uterine myometrium. This condition carries a high risk of massive hemorrhage, uterine rupture, and potentially life-threatening complications. **Methods:** We present a case of a 35-year-old patient who underwent in vitro fertilization (IVF) and was diagnosed with an IMP located in the back-isthmian portion of the uterus by ultrasound scan. **Results:** We performed a conservative treatment approach based on the gestational sac location and the patient’s stable clinical condition and desire for future fertility. We first administered mifepristone 600 mg, followed by intracavitary methotrexate under ultrasound guidance. Although originally planned, a uterine artery embolization was not performed due to the evidence of bilateral anastomoses between the uterine and ovarian arteries. Progressive reabsorption of pregnancy was observed over the course of 8 months. **Conclusions:** Non-surgical management can be considered for IMP, thus allowing fertility preservation.

## 1. Introduction

Intramural pregnancy (IMP), reported in approximately 0.1% of ectopic pregnancies, is an unusual and extremely rare type of ectopic pregnancy [1]. In IMP, the pregnancy is located within the uterine wall, surrounded by the myometrium, and separated from the uterine cavity, fallopian tube, and round ligament [2].

Risk factors for IMP are curettage (37%), cesarean section (11%), salpingectomy (12%), assisted reproductive techniques with embryo transfer (12%), myomectomy (10%), and adenomyosis (5%) [3].

IMP can present with a range of non-specific signs and symptoms, with vaginal bleeding and lower abdominal pain being the most common initial clinical manifestations [3]. In some cases, IMP can also manifest with acute abdominal pain or hemorrhagic shock due to uterine rupture [3].

Prognosis of IMP is generally poor due to the high risk of complications. Most cases are diagnosed and managed in the first trimester, as progression beyond 12 weeks is rare. However, there have been a few reported cases of IMP continuing into the second trimester. A gestational age >10 weeks and a gestational sac located in the uterine fundus are associated with a 7–8 times higher risk of uterine rupture compared to an earlier gestational age and different gestational sac location [4].

The diagnosis of IMP is complex and requires the visualization of trophoblastic invasion into the myometrium. This can be achieved through ultrasonography or magnetic resonance imaging (MRI) [5]. Additionally, power Doppler imaging can be useful in diagnosis, as it highlights the characteristic peripheral slow blood flow associated with trophoblastic tissue [1]. Also, 3D sonography ensures a more accurate localization of the gestational sac in relation to the uterine corn, the interstitium, and the endometrial cavity compared to 2D sonography [2,6].

Management of IMP should be personalized, based on gestational sac location, gestational age at diagnosis, the patient’s clinical condition, and desire to maintain fertility [1,3]. The treatment is not standardized, and the options include expectant management, systemic intramuscular injection of methotrexate (MTX), ultrasound-guided injection of MTX and/or injection of potassium chloride into the gestational sac, uterine artery embolization (UAE), and surgery [7]. The approach to IMP shares similarities with cesarean scar pregnancy, particularly regarding the use of UAE. The experience accumulated in managing scar pregnancy has demonstrated the effectiveness of UAE in reducing blood flow to the pathological implantation site, thereby reducing the risk of hemorrhage and facilitating conservative treatment.

## 2. Patient and Methods

This is a case report of a 35-year-old woman with an intramural isthmic pregnancy following IVF. Diagnosis was based on transvaginal and 3D ultrasounds, supported by power Doppler imaging and serial beta-hCG measurements.

Treatment included 600 mg oral mifepristone, followed by 75 mcg methotrexate (MTX) injected locally into the gestational sac under ultrasound guidance. Although initially planned, a UAE was not performed due to the bilateral uterine–ovarian anastomoses observed on angiography. Clinical follow-up involved ultrasound and beta-hCG monitoring over three months, with hysteroscopic evaluation at 3 and 8 months.

No new methods or codes were used. Data are available from the authors upon request.

Ethical statements: 

Animal studies: No animal studies are presented in this manuscript.

Human studies: Ethical approval was not required as this is a retrospective analysis of anonymized clinical data. No experimental interventions were performed. The study complied with local legislation and institutional requirements. Written informed consent was obtained.

Identifiable human data: Written informed consent was obtained for the publication of any potentially identifiable data.

Data availability: Raw data are available from the authors without undue reservation.

Generative AI disclosure: No generative AI was used in the preparation of this manuscript.

## 3. Patient Information

A 35-year-old female, pregnant for the first time through intracytoplasmic sperm injection (ICSI), was referred to our Obstetrics and Gynecology Department for a suspicion of cervical pregnancy at 6^6/7^ gestational weeks. At the time of referral, the patient was asymptomatic.

Before being referred to our institution, the patient had undergone two initial β-hCG assessments at the IVF center: the first one measured at 4 + 1 weeks of gestation was 180 mUI/mL, and the second one—performed eight days later—showed a rising trend. Despite the abnormal finding, an additional eight days passed before the patient was referred to our hospital, where a β-hCG level of 23,434 mUI/mL was recorded at admission.

**Figure 1:**
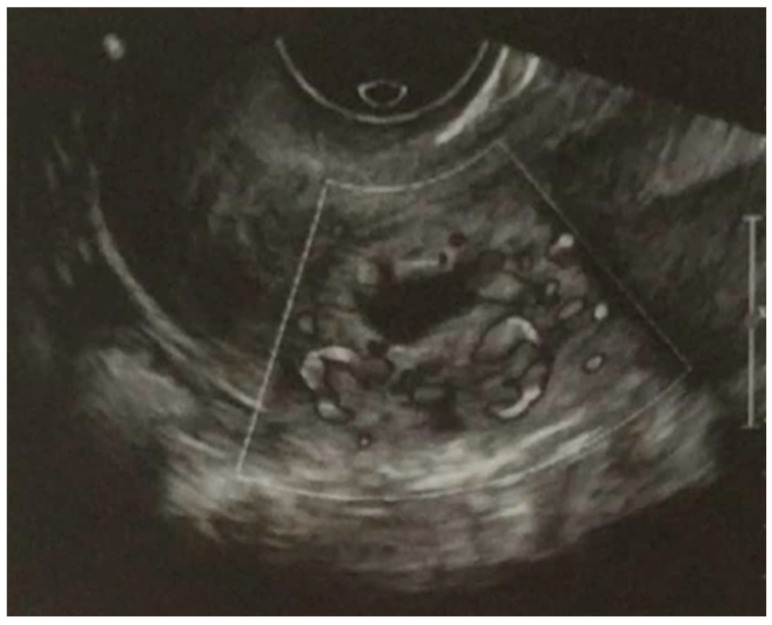
Ultrasound image before the start of treatment.

The sonography assessment (Figure 1) revealed a gestational sac located in the posterior wall of the uterine isthmus with no connection with the uterine cavity. A yolk sac was observed within the sac, along with echoes suggestive of an embryo, with no detectable cardiac activity.

The patient was scheduled for a follow-up scan one week later, when the diagnosis of IMP was confirmed. Specifically, the scan revealed a gestational sac 31 × 24 × 31 mm in size, with a rich peripheral and inner vascularization, and a myometrium thickness between the sac and the posterior uterine wall of 1.5 mm. No free fluid in the Douglas’s pouch or in the abdominal cavity was identified.

After the diagnosis, we counseled the patient about potential treatment options.

Despite the β-hCG level exceeding the typical threshold for MTX treatment—commonly set between 3000 and 5000 mUI/mL—we offered a conservative approach, including UAE with an MTX injection preceded by 600 mg of mifepristone orally, which the patient accepted. This decision was based on the patient’s stable clinical condition, the absence of embryonic cardiac activity, and the location of the gestational sac. Of note, UAE combined with MTX administration is routinely used at our institution as a fertility-preserving strategy in cases of cesarean scar pregnancies.

After the mifepristone administration, the patient remained asymptomatic. We then performed an angiography (20 h after mifepristone), which revealed bilateral anastomoses between the uterine and ovarian arteries, thus making a UAE a potentially dangerous procedure. Therefore, we decided to skip the UAE and administered MTX (75 mg) intracavitary into the gestational sac under ultrasound guidance. After the procedure, the patient experienced significant abdominal pain in the periumbilical region, which responded to analgesic therapy. An episode of vaginal bleeding occurred a few days later, and then she remained asymptomatic and was discharged on day four after the procedure. On the day of discharge, six days after the mifepristone administration, her beta-hCG level was 26,178 mUI/mL, and a transvaginal ultrasound confirmed the reduction in size of the gestational sac with no signs of complications.

**Figure 2:**
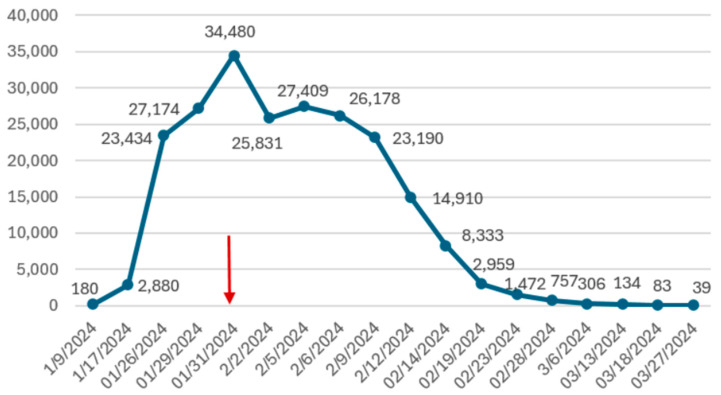
Beta-hCG trend. Red arrow indicates the day of mifepristone administration. Beta-hCG are expressed as mUI/mL.

During follow-up visits on days 3, 8, and 13 after discharge, the patient remained asymptomatic. At each visit, a transvaginal ultrasound was performed, documenting progressive involution of the gestational sac. A corresponding decline in beta-hCG levels was also observed (Figure 2).

Ultrasound examinations showed a hypotonic sac with no embryonic echoes and a no longer visible yolk sac.

**Figure 3:**
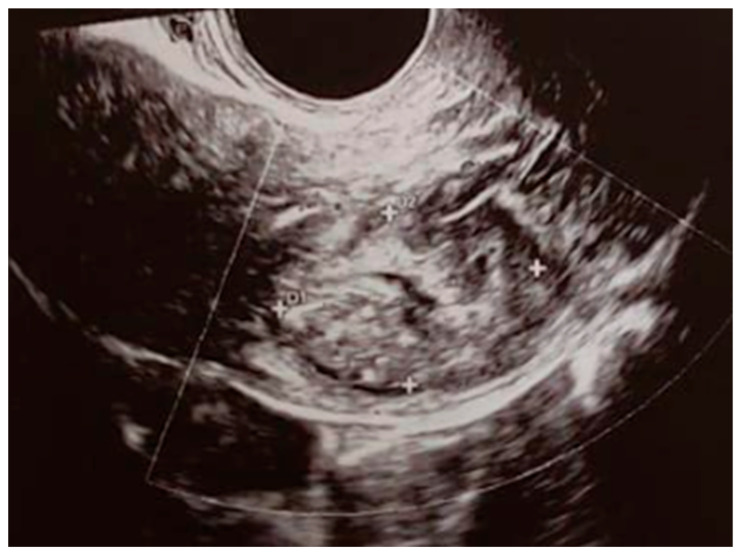
Ultrasound image taken 17 days post-treatment.

Seventeen days after MTX therapy, the patient presented to our emergency room with a fever (fastigium 38.4 °C) and pelvic pain. Vaginal swabs, blood cultures, blood tests, and an ultrasound (Figure 3) were performed, and empirical antibiotic therapy was initiated. After two days, the fever subsided, and the patient was discharged on the fourth day. As all microbiological tests came back negative, the fever was hypothesized to be related to cytolysis caused by the treatment. An MRI was not performed since the clinical presentation, laboratory findings, and the ultrasound examination were not suggestive of complications, such as abscesses or hemorrhages. Additionally, the rapid resolution of symptoms with empirical therapy supported the hypothesis of a self-limiting inflammatory response rather than an underlying complication.

**Figure 4:**
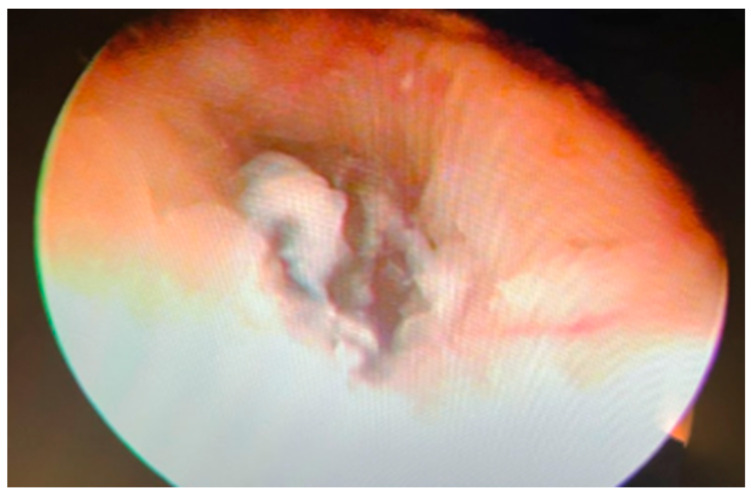
Hysteroscopy after 3 months allows identification of the ostium of the conduit located in the posterior wall of the cervix.

After three months, the patient underwent a hysteroscopy. A conduit was identified on the posterior cervical wall, just before the internal uterine os, suggestive of the IMP location (Figure 4).

An additional ultrasound and hysteroscopic examination were performed after eight months. We observed a non-patent opening at the site of the previous ectopic pregnancy. The cervix and uterine cavity appeared normal. The ultrasound revealed a posterior isthmic area with a residual mass measuring 10 × 5 mm, which had decreased in size compared to the previous examination.

## 4. Discussion

IMP, occurring when the gestational sac implants within the myometrium, is an extremely rare condition, with an incidence of less than 1% of all ectopic pregnancies. Only about 70 cases have been reported so far in the literature [4,8]. The rarity of these cases makes both diagnosis and management particularly challenging [2].

The growing prevalence of risk factors for IMP, such as assisted reproductive techniques, uterine surgeries (e.g., cesarean sections and myomectomies), and other types of uterine trauma (such as dilatation and curettage), is expected to raise the occurrence of this ectopic pregnancy [3,4,9]. Assisted reproductive technologies, particularly IVF and embryo transfer, are significant contributors to IMP. Errors in embryo placement and the changes in implantation mechanisms associated with these procedures may facilitate abnormal implantation within the myometrium [8,9]. Additionally, uterine surgeries like cesarean sections and myomectomies can create false tracts in the myometrium, allowing embryos to implant in these abnormal sites [4,8]. These factors underscore the need for heightened awareness and early diagnosis to prevent severe complications [4].

Ultrasound plays a vital role in diagnosing IMP, providing a clearer visualization of the gestational sac’s location relative to the uterine cavity, which aids in distinguishing it from other ectopic pregnancies. All women with known risk factors for ectopic pregnancies should perform the first obstetric ultrasound earlier than what is recommended for pregnancy dating. A delay in the diagnosis of all types of ectopic pregnancy puts the patient at risk of hemorrhage, uterine rupture, and, in the most severe cases, needing an hysterectomy. Also, late diagnosis limits treatment options due to the increased size of the gestational sac and the embryo.

One key element of this case is the timeliness of the diagnosis, which allowed a prompt intervention and, in turn, a favorable outcome [8,10].

Despite the rarity of IMP, there is no standardized treatment in the literature.

The decision-making process is highly dependent on factors such as the patient’s clinical condition, ultrasound findings (gestational sac size and location, presence of an embryo and its cardiac activity, and gestational age), and reproductive goals [1,3]. For women without a childbearing desire, a surgical treatment can be appropriate [11]. In turn, for women wishing to preserve fertility, a personalized and less invasive treatment strategy is paramount. In our case, the patient’s desire to preserve fertility was a key factor in choosing a medical approach. Notably, the current literature supports the use of medical management as a first-line treatment for stable IMP cases, particularly when the pregnancy is well-localized, and there is no evidence of complications [6,7]. Also, although being mini-invasive for the patient, hysteroscopic surgery, dilatation and curettage, or laparoscopy can damage the uterine lining and possibly affect future fertility, as well as increase the risk of uterine rupture in subsequent pregnancies.

The role of UAE in IMP management, as demonstrated in previous reports on scar pregnancies, has shown promise in reducing the risk of uterine rupture by halting the growth of the ectopic pregnancy [12]. Although the evidence for its use in IMP is still limited, the UAE can be considered a practical option for preserving the uterus, especially in tertiary centers where such procedures are available. In addition, UAE may be used as an adjunct to MTX therapy, improving overall outcomes and reducing the need for more invasive surgical options. This approach also holds value in anticipation of elective surgery, as it helps to preserve uterine morphology, reduces blood loss at surgery, and supports better outcomes in subsequent pregnancies [12].

Regarding MTX use, its success varies based on gestational sac size, location, and vascularization, with smaller, well-localized cases showing better responses. As the complexity of the case increases (i.e., larger gestational sacs and more advanced gestational age), the likelihood of success with MTX alone decreases, prompting the need for alternative or adjunctive measures. In this context, the combination of MTX and UAE can be particularly advantageous. In addition, the combined approach can improve outcomes by addressing both the medical and vascular aspects of the condition [12]. Our experience supports the use of systemic or local administration of MTX followed by expectant management as an effective non-invasive treatment strategy for IMP.

A strength of our case was the multidisciplinary approach and the ability to adapt the treatment to the patient’s unique anatomy. As seen here, atypical vascular structures, including bilateral uterine–ovarian artery anastomoses, can contraindicate UAE. This underscores the importance of a comprehensive pre-procedural assessment and the flexibility to modify the treatment plan to ensure optimal management despite anatomical challenges.

Our case underlines the importance of referring IMP cases to tertiary centers where all potential treatment options can be pursued [13] according to the specific characteristics of the case and allowing a patient-centered approach.

## 5. Conclusions

The management of IMP requires an individualized approach that takes into consideration clinical factors, ultrasound findings, the patient’s characteristics, and reproductive goals. An early ultrasound screening is pivotal for a timely diagnosis and intervention, thus preventing potential life-threatening complications, such as hemorrhage and uterine rupture. The choice of treatment—combining medical management (local MTX), UAE, and elective minimally invasive surgical options—should be tailored to the complexity of each case to achieve the best outcome for the patient. Given the rarity of the IMP condition, our case offers valuable insights for the clinical community.

## Data Availability

The data supporting the findings of this study are available from the corresponding author upon reasonable request. No publicly archived datasets were generated or analyzed in this study due to the nature of the clinical case and privacy considerations.

## Abbreviations

The following abbreviations are used in this manuscript:

IMP	Intramural pregnancy
EP	Ectopic pregnancy
MTX	Methotrexate
UAE	Uterine artery embolization
IVF	In vitro fertilization
MRI	Magnetic resonance imaging
HCG/β-HCG	Human chorionic gonadotropin (beta subunit)
ICSI	Intracytoplasmic sperm injection
